# A novel essential protein identification method based on PPI networks and gene expression data

**DOI:** 10.1186/s12859-021-04175-8

**Published:** 2021-05-13

**Authors:** Jiancheng Zhong, Chao Tang, Wei Peng, Minzhu Xie, Yusui Sun, Qiang Tang, Qiu Xiao, Jiahong Yang

**Affiliations:** 1 School of Information Science and Engineering, Hunan Normal University, Changsha, 410081 China; 2 Hunan Provincial Key Lab on Bioinformatics, School of Computer Science and Engineering, Hunan Provincial Key Laboratory of Intelligent Computing and Language Information Processing, Changsha, 410083 China; 3 College of Information Engineering and Automation, Kunming University of Science and Technology, Kunming, 650500 Yunnan China; 4 College of Engineering and Design, Hunan Normal University, Changsha, 410081 China

**Keywords:** Essential proteins, The PPI networks, Jaccard similarity index, Edge clustering coefficient

## Abstract

**Background:**

Some proposed methods for identifying essential proteins have better results by using biological information. Gene expression data is generally used to identify essential proteins. However, gene expression data is prone to fluctuations, which may affect the accuracy of essential protein identification. Therefore, we propose an essential protein identification method based on gene expression and the PPI network data to calculate the similarity of "active" and "inactive" state of gene expression in a cluster of the PPI network. Our experiments show that the method can improve the accuracy in predicting essential proteins.

**Results:**

In this paper, we propose a new measure named JDC, which is based on the PPI network data and gene expression data. The JDC method offers a dynamic threshold method to binarize gene expression data. After that, it combines the degree centrality and Jaccard similarity index to calculate the JDC score for each protein in the PPI network. We benchmark the JDC method on four organisms respectively, and evaluate our method by using ROC analysis, modular analysis, jackknife analysis, overlapping analysis, top analysis, and accuracy analysis. The results show that the performance of JDC is better than DC, IC, EC, SC, BC, CC, NC, PeC, and WDC. We compare JDC with both NF-PIN and TS-PIN methods, which predict essential proteins through active PPI networks constructed from dynamic gene expression.

**Conclusions:**

We demonstrate that the new centrality measure, JDC, is more efficient than state-of-the-art prediction methods with same input. The main ideas behind JDC are as follows: (1) Essential proteins are generally densely connected clusters in the PPI network. (2) Binarizing gene expression data can screen out fluctuations in gene expression profiles. (3) The essentiality of the protein depends on the similarity of "active" and "inactive" state of gene expression in a cluster of the PPI network.

## Background

Proteins are generally involved in the life activities of organisms. Essential proteins are often found in protein complexes. Loss of essential proteins could cause lethality and even lead to the inability of the body to survive [1, 2].

Therefore, the identification of essential proteins not only helps us understand the minimal requirements for cell life but also plays a vital role in the discovery of human disease genes. Various experimental methods are used to identify essential proteins, such as a single gene knockout [3], RNA interference [4], and conditional knockouts [5].

Although experimental methods have achieved excellent results, it still has some shortcomings such as time-consuming and expensive. Nowadays, a variety of biological data have been generating rapidly by high-throughput experimental technologies, such as genomics, transcriptomics, and proteomics datasets. For researchers, it has become possible to identify essential proteins with computational methods. The computational methods can be classified into two categories: unsupervised and supervised machine learning methods.

Unsupervised methods usually identify essential proteins based on some essentiality-related data, including the PPI networks, cellular localization data, and gene expressing data, etc. As for the topological of the PPI network, various prediction models based on the centrality-lethality rule are proposed. Because essential proteins in the PPI network are more likely to be hubs nodes, and elimination of hubs nodes may cause the PPI network to break down. Various centrality measures for prediction of essential proteins include Degree Centrality (DC) [6], Betweenness Centrality (BC) [7], Closeness Centrality (CC) [8], Subgraph Centrality (SC) [9], Eigenvector Centrality (EC) [10], Information Centrality (IC) [11]. However, these measures only consider the topological features of the PPI network and ignore false positives of the PPI network. Some researchers adopt biological information to eliminate the effect of false-positive data on the PPI network. Li and Tang et al. propose essential protein prediction methods called PeC and WDC by combining the PPI network and gene expression information [12, 13]. Compared with non-essential proteins, essential proteins tend to be conserved. According to this observation, Peng et al. adopt the orthology and PPI networks to predict essential proteins [14]. Li et al. propose an identification method,SON, by using the information of subcellular localization, orthologous proteins and PPI networks [15]. Li et al. utilize an Extended Pareto Optimality Consensus model to find the triangular structure in the PPI network and combine the orthology information for the prediction of essential proteins [16].Based on prior knowledge, Li et al. propose two essential protein identification algorithms, CPPK and CEPPK [17]. Li et al. propose a new prediction method for evaluating the confidence of each interaction in PPI network to infer essential proteins [18]. Based on overlapping essential modules, Zhao et al. adopt gene expression profiles to predict essential proteins [19].

With the generation and improvement of multi-omics data, it has become possible to construct comprehensive dynamic networks to identify essential proteins. For predicting essential proteins better, Lichtenberg et al. build a time series dynamic network by combining gene expression data at different time points and the protein interactions data [20]. Xiao et al. propose a prediction method by constructing NF-PIN dynamic network using the time series model and 3_sigma principle to filter out the noise of gene expression [21]. Recently, Li et al. construct TS-PIN dynamic network by combining gene expression profile and subcellular localization information to predict essential proteins [22]. Li et al. introduce a sub-network partition method to predict essential proteins by using the subcellular localization information [23]. Fan et al. adopt an improved PageRank algorithm to identify essential proteins based on gene expression and subcellular localization information [24]. Lei et al. incorporate the multiple biological characteristics, including PPI network, GO annotation data, subcellular localization information, and protein complexes information, to identify essential proteins by using random walk algorithms [25]. Zhang et al. propose a method to predict essential proteins by fusing dynamic PPI networks [26].Li et al. identify essential proteins by computing each protein's topology potential [27]. Peng et al. propose the UDoNC method to predict the essential proteins [28].

On the other hand, some prediction methods adopt supervised learning methods and use machine learning algorithms to identify essential proteins, such as SVM, Random Tree, RBF network, and Naïve Bayes. Gustafson et al. propose using Naïve Bayes to identify essential proteins based on gene expression data and topological features in the PPI network [29]. Compared with unsupervised methods, the performance of supervised methods for detecting essential proteins are often better than that of unsupervised methods. Hwang et al. construct an SVM classifier by using some biological features (such as ORF, ST, PHY) and some topological features (such as DC, BD, CC) of the PPI network [30]. Zhong et al. adopt the GEP method and an XGBFEMF framework to predict the essential proteins [31, 32]. Deng et al. predict essential proteins by combining Naïve Bayes classifier, C4.5 decision tree, CN2 rule, and logistical regression model [33]. Kim et al. adopt machine learning methods to predict essential proteins by using topological properties in the GO-pruned PPI network [34]. Recently, Zeng et al. design a deep learning framework for the prediction of essential proteins [35].

The methods based on PPI network and gene expression data may, to some extent, eliminate false positive and false negative of protein interaction data. However, the gene expression profile is a set of values with large fluctuations that may affect prediction performance. When studying complex biological systems, Niehrs et al. point out that the "on" and "off" of genes at different times played an important role in biological development [36]. To introduce the "on" and "off" of states of genes, we propose an essential protein prediction method, named JDC, based on the PPI data and gene expression data by using the essential Degree Centrality with Jaccard similarity index. JDC can eliminate the fluctuations of gene expression data by calculating the similarity of "active" and "inactive" state of gene expression in a cluster of the PPI network. Compared with the state-of-the-art methods on four organisms, our method is more accurate and has higher specificity and sensitivity.

## Methods

### Overview

**Fig. 1:**
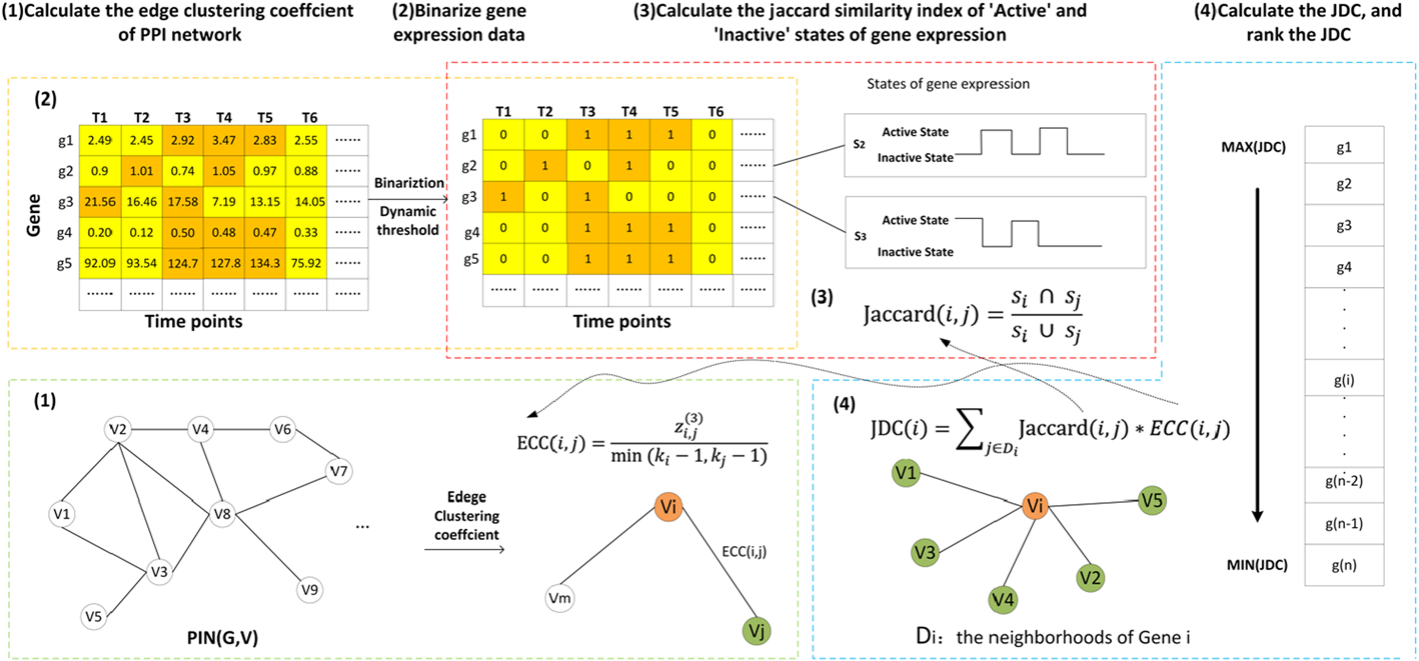
An illustration of JDC

Figure 1 illustrates an example of JDC to predict essential proteins. The JDC algorithm incorporates gene expression information with PPI network data. The whole process of JDC includes the following steps.(1) ECC is used to characterize the probability of two proteins being in a cluster from a topology perspective (2) A dynamic threshold is set to binarize gene expression data for filtering out the fluctuations in gene expression profiles. (3) The Jaccard similarity index measures the similarity of two proteins that has the “active” and “inactive” state of gene expression profiles; (4) JDC scores are calculated by integrating the ECC values and Jaccard similarity index. According to those steps, we use top rank analysis in the JDC value to verify the performance of our method.

### Experimental datasets

We have collected the four organisms: Saccharomyces cerevisiae (Bakers’ Yeast), Escherichia coli (E.coli), Drosophila melanogaster (Fly), and Homo sapiens (Human) to evaluate the JDC method.

The PPI data of Yeast and E.coli were obtained from the DIP database. The PPI network of E. coli has 2727 proteins and 11,803 edges after filtering the self-interactions and the repeated interactions. There were 5093 proteins and 24,743 edges in the PPI network of Yeast. The PPI data of Fly and Human can be downloaded from the BioGRID database. There were 76,480 edges and 9217 nodes in Fly datasets, and the 504,848 edges and 18,009 nodes in Human datasets. By converting the id and filtering the self-interactions and the repeated interactions, there were 37,992 edges and 6481 nodes in Fly network, and 348,871 edges and 15,721 nodes in Human network.

Essential proteins were integrated by the four databases of MIPS [37], SGD [38], DEG [39], and SGDP [40]. There are 1167 essential proteins present in Yeast PPI network. Out of all 2727 proteins in the E.coli network, 254 were essential. The essential proteins of Fly and Human can be obtained from the OGEE database. There are 408 essential proteins and 13,373 non-essential proteins in Fly datasets. The number of essential genes human was 7123.

The Gene Expression data were downloaded from the NCBI Gene Expression Omnibus website. After pretreatment and normalization, 6777 Yeast gene products and 36 samples were obtained. Similarly, the gene expression data of E.coli was also downloaded from this website. After removing the redundant data, the E.coli gene expression data had 7312 genes and 8 samples. GSE67547 is the gene-expression profiles of Fly with 11,952 genes and 66 samples, whereas GSE86354 is the human tissue-specific RNA-seq expression profiling by high throughput sequencing.

### Edge clustering coefficient (ECC)

Radicchi et al. first propose the edge clustering coefficient that is an important topological feature in computational networks [41]. Wang et al. adopt the edge clustering coefficient to predict essential proteins in the yeast PPI network, which also has achieved a good detection effect [42]. The advantage of the edge clustering coefficient is to describe the clustering characteristics of PPI networks from the perspective of topology. We adopt the ECC shown in formula (1) for our method to calculate the topological attribute of the two nodes, *i* and *j*:

1 $$\mathrm{ECC}\left(i,j\right)=\frac{{z}_{i,j}^{(3)}}{\mathrm{min}({k}_{i}-1,{k}_{j}-1)}$$

where ${z}_{i,j}^{(3)}$ denotes the number of actual triangles formed by the edge $\left(i,j\right)$ in PPI networks, then, the number of possible triangles determined by the minimum degree of node *i* and *j* is defined as $\mathrm{min}({k}_{i}-1,{k}_{j}-1)$. ECC is used to describe how tightly two proteins are connected. The larger the ECC value is, the more likely two connected proteins are in the same cluster. Thus, the PPI network was divided into multiple clusters by calculating the ECC value of each pair of interacting proteins.

### Binarization of gene expression data

Gene expression data are continuous and produced from microarray experiments. However, the gene expression from high-throughput experiments are prone to large fluctuations. Sahoo et al. performed a Boolean analysis of mouse B cell gene expression data to understand gene regulation and gene function [43]. In order to eliminate fluctuation of gene expression, in this paper, we use a threshold strategy to covert the continuous values to the discrete state values, and then characterize gene expression data with "active" and "inactive" state.

In this paper, we select one sigma value close to the mean value as the threshold for screening the “active” and “inactive" state of gene expressions. Formula (2) is the mean of gene expression data. Formula (3) is the standard deviation of gene expression, and Formula (4) is the volatility of gene expression. The threshold parameter is defined in Formula (5).

2 $$U\left(i\right)=\frac{{\sum }_{t=1}^{n}{E}_{t}^{\left(i\right)}}{n}$$

3 $${\sigma }^{2}\left(i\right)=\frac{{\sum }_{t=1}^{n}{\left(U\left(i\right)-{E}_{t}^{\left(i\right)}\right)}^{2}}{n}$$

4 $$V\left(i\right)=\frac{1}{1+{\sigma }^{2}\left(i\right)} (4)$$

5 $$G\left(i\right)=U\left(i\right)+2*\sigma \left(i\right)*V\left(i\right)$$

where ${E}_{t}^{(i)}$ is the expression value of protein *i* at time point *t*, $U\left(i\right)$ is the mean of expression value of protein *i*, $\sigma \left(i\right)$ is the standard deviation of expression data of protein *i*, $V\left(i\right)$ is the volatility of expression value of protein *i*, $G\left(i\right)$ is the threshold parameter of expression value of protein *i*.

$G$ denotes a matrix constructed from gene expression data, $N$ is the number of genes, and $M$ is the time of proteins:

6 $$S=\left(\begin{array}{ccc}{s}_{11}& \cdots & {s}_{1M}\\ \vdots & \ddots & \vdots \\ {s}_{N1}& \cdots & {s}_{NM}\end{array}\right)$$

where ${s}_{i,t}$ is the expression level of protein *i* at time *t*. If the expression value of ${s}_{i,t}$ is higher than the specified threshold, the "active" gene expression is defined as "1". If the value of ${s}_{i,t}$ is not higher than the specified threshold $G\left(i\right)$, it is "inactive" gene expression and defined as "0". The calculation formula is as follows:

7 $${s}_{i,t}^{^{\prime}}=\left\{\begin{array}{c}1,{ s}_{i,t}>G\left(i\right)\\ 0,{ s}_{i,t}\le G\left(i\right)\end{array} \right.$$

where ${s}_{i,t}^{^{\prime}}$ is the activity of protein i at time t. $S$ is updated to the matrix with Boolean values. In this paper, the gene expression data are transformed into Boolean values that can reflect the "active" and "inactive" state of gene expression.

### Jaccard similarity index

The Jaccard coefficient is generally used to measure the similarity of two discrete objects. Numanagic et al. proposed the SEDEF framework based on the Jaccard coefficient, which can accurately predict segmental duplications (SDs) [44]. Wallace et al. introduced the Jaccard coefficient into the prediction of disease-disease relationship and deduced the information of the interaction network [45]. In this paper, we compare the co-expression of two different related proteins with the Jaccard coefficient. Therefore, the Jaccard coefficient of edge $\left(i,j\right)$ can be defined as:

8 $$\mathrm{Jaccard}\left(i,j\right)=\frac{{S}_{i} \cap {S}_{j}}{{S}_{i} \cup {S}_{j}}$$

where ${S}_{i}$ and ${S}_{j}$ represent the Boolean values of the gene expression data of gene *i* and gene *j*. The Jaccard correlation coefficient should be between 0 and 1. Here, we define the value as the similarity of active expression between gene *i* and gene *j* in a cluster of PPI networks.

### JDC measure index

It has been proved that genes with similar functions often exhibit similar expression patterns, known as the "guilt-by-association" principle [46]. Based on the edge clustering coefficient (ECC) and Jaccard coefficient (Jaccard), we propose a new measurement method with Jaccard similarity index (JDC), which is named as the essential Degree Centrality. We describe the clustering degree of two proteins from topological and biological perspectives. Therefore, we define the clustering degree of an edge $(i, j)$ in the PPI network as follows:

9 $${J}_{c}\left(i,j\right)=Jaccard\left(i,j\right)*ECC\left(i,j\right)$$

For protein *i*, we define its JDC value as the sum of the probability that the protein and its neighbors belong to the same cluster:

10 $$\mathrm{JDC}\left(i\right)={\sum }_{j\in {D}_{i}}Jaccard\left(i,j\right)*ECC(i,j)$$

where ${D}_{i}$ denotes all the neighborhoods of node *i*. Then, the node i and the neighbors are divided into a cluster. The values measured by JDC depend on the similarity of "active" and "inactive" state of gene expression in a cluster of PPI networks.

In this paper, we propose an essential protein identification method based on PPI data and gene expression. The advantage of this method is that the calculation is simple, and the performance of JDC is better than some state-of-the-art prediction methods.

## Results

### ROC curves and its AUC analysis

**Fig. 2:**
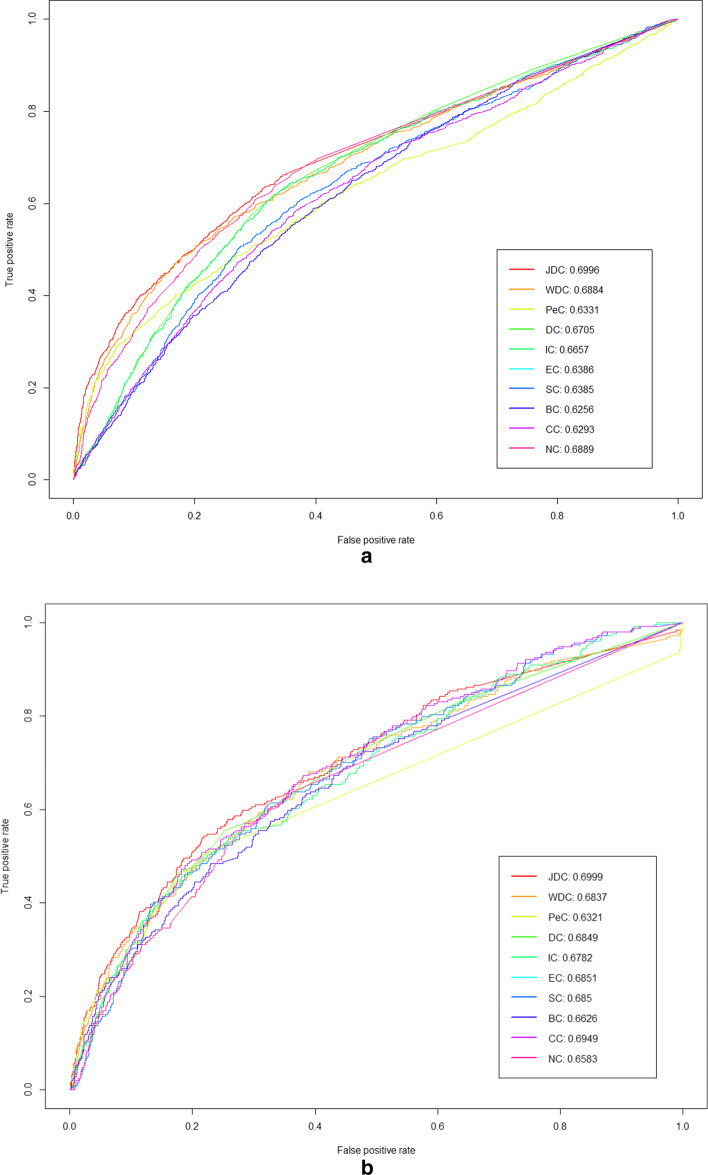
ROC curves and AUC values of the JDC method and other methods using the individual features. **a **Yeast data. **b **E.coli data

In this section, we adopt receiver operating characteristic (ROC) curves to evaluate the global performance of each method. The comparison results are shown in Fig. 2.

As shown in Fig. 2, the ROC curve of JDC is almost above that of other prediction methods. The area under the ROC curve (AUC) on both two datasets are 0.6996, and 0.6999 respectively, which are the highest values among all methods. The ROC results obtained by ten methods demonstrate that JDC is more suitable for predicting essential proteins.

To show that our method has better performance, we focus on comparing JDC with WDC and PeC, because these methods use the same input data. Li and Tang have introduced the Pearson correlation coefficient to weight PPI network based on ECC, which effectively reduced false positives and false negatives in PPI network on Yeast data [12, 13]. Compared with those methods, JDC not only takes the false positive and false negative data into consideration on PPI data, but also introduces the "active" and "inactive" states of gene expression. The AUC of JDC method on the yeast dataset improves more 0.0112 and 0.0665 than that of WDC and Pec, respectively. The similar results are obtained in the experimental results of E.coli dataset.

The advantage of introducing different states is to eliminate fluctuations in gene expression data, especially between two genes, the expression value of one gene is particularly high, and thus affects the similarity value. JDC can fully consider the co-expression state of the connected genes at multiple different moments, while WDC and Pec compare the similarity of the specific expression values of the two genes at different times.

**Fig. 3:**
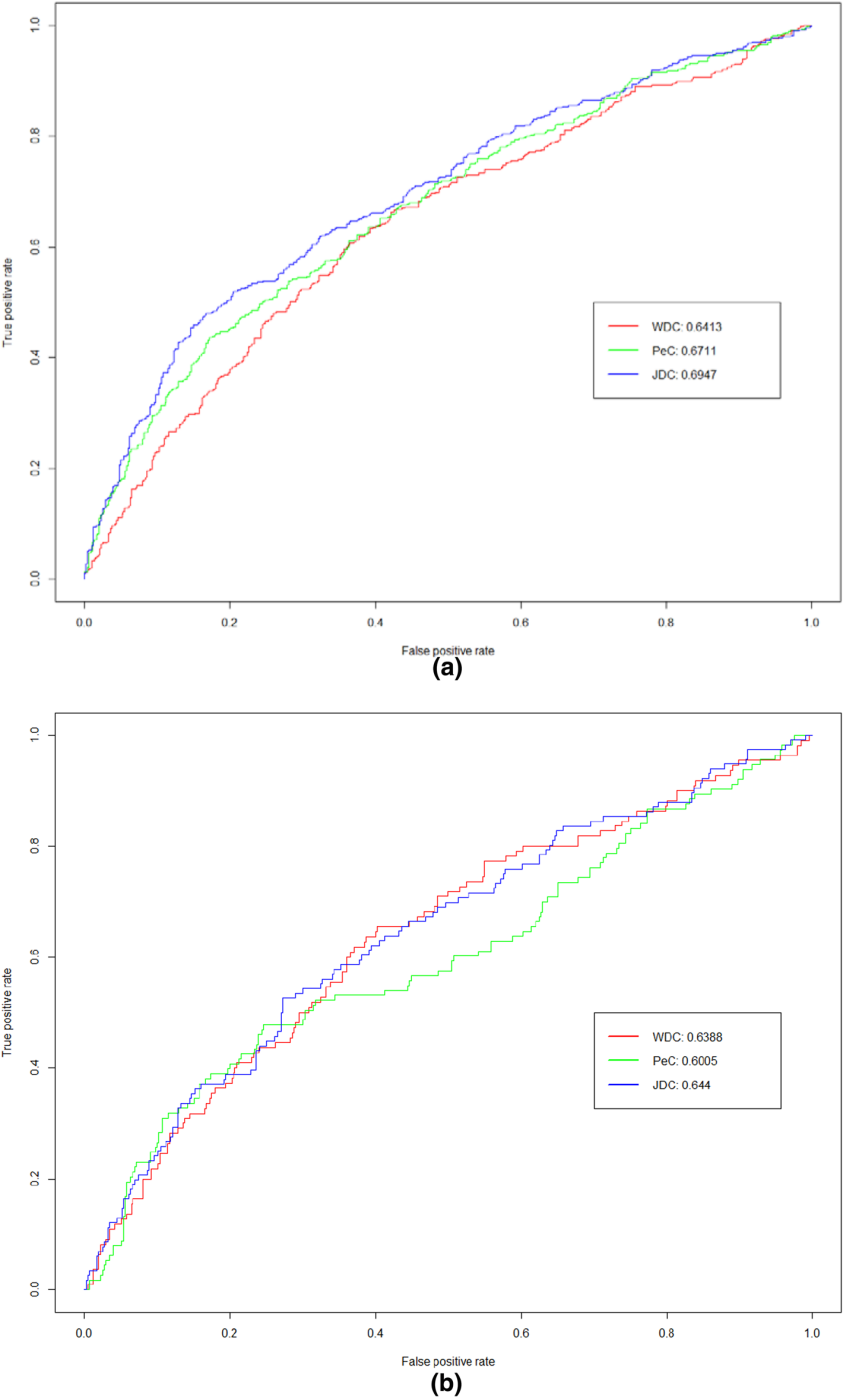
ROC curves and AUC values of the JDC method and other methods using the individual features in the top 20% ranked proteins. **a **Yeast data. **b ** E.coli data

To further compare the performance of JDC, WDC and Pec, we analyze the ROC curve based on the top 20% of proteins ranked by each method. The ROC curves are shown in Fig. 3. As can be seen from Fig. 3, the AUC of JDC is higher than that of WDC and PeC both on yeast and E.coli datasets.

### Accuracy analysis

Where denotes the number of true-positive proteins, denotes the number of false-positive proteins, denotes the number of true negative proteins, and denotes the number of false-negative proteins. In this paper, true-positive is that real essential proteins are correctly predicted as essential proteins, false positive is that non-essential proteins are predicted as essential proteins, true negative is that non-essential proteins are correctly predicted as non-essential proteins, and false negative is that the essential proteins are predicted as non-essential proteins. The results on Yeast and E.coli data are in Table 1.

The Formula (11)–Formula (17) are as follows:

11 $$SN=\frac{TP}{TP+FN}$$

12 $$SP=\frac{TN}{TN+FP}$$

13 $$FPR=\frac{FP}{TN+FP}$$

14 $$PPV=\frac{TP}{TP+FP}$$

15 $$F-measure=\frac{2*TP}{2*TP+FP+FN}$$

16 $$ACCuracy=\frac{TP+TN}{TP+TN+FP+FN}$$

17 $$MCC=\frac{TP*TN-FP*FN}{\sqrt{\left(TP+FP\right)*\left(TP+FN\right)*\left(TN+FP\right)*\left(TN+FN\right)}}$$

where $\mathrm{TP}$ denotes the number of true-positive proteins, $FP$ denotes the number of false-positive proteins, $TN$ denotes the number of true negative proteins, and $FN$ denotes the number of false-negative proteins. In this paper, true-positive is that real essential proteins are correctly predicted as essential proteins, false positive is that non-essential proteins are predicted as essential proteins, true negative is that non-essential proteins are correctly predicted as non-essential proteins, and false negative is that the essential proteins are predicted as non-essential proteins. The results on Yeast and E.coli data are in Table 1.

It can be seen from Table 1 that the values of $SN$, $SP$, $PPV$, $NPV$, $F-measure$, $ACC$, and $MCC$ of JDC on Yeast data are 0.4604, 0.8403, 0.4604, 0.8403, 0.4604, 0.7535 and 0.3007 respectively. Each evaluation criterion for JDC is better than other prediction methods. Meanwhile, the values of $SN$, $SP$, $PPV$, $NPV$, $F-measure$, $ACC$ and $MCC$ of JDC on E.coli data are 0.2835, 0.9264, 0.2835, 0.9264, 0.2835, 0.8665 and 0.2099 respectively, which outperforms all other methods listed in Table 1. The lower the $FPR$, the better the method. The $FPR$ value of JDC is also the lowest of all methods in the two data sets.

### Top analysis and overlapping analysis

**Fig. 4:**
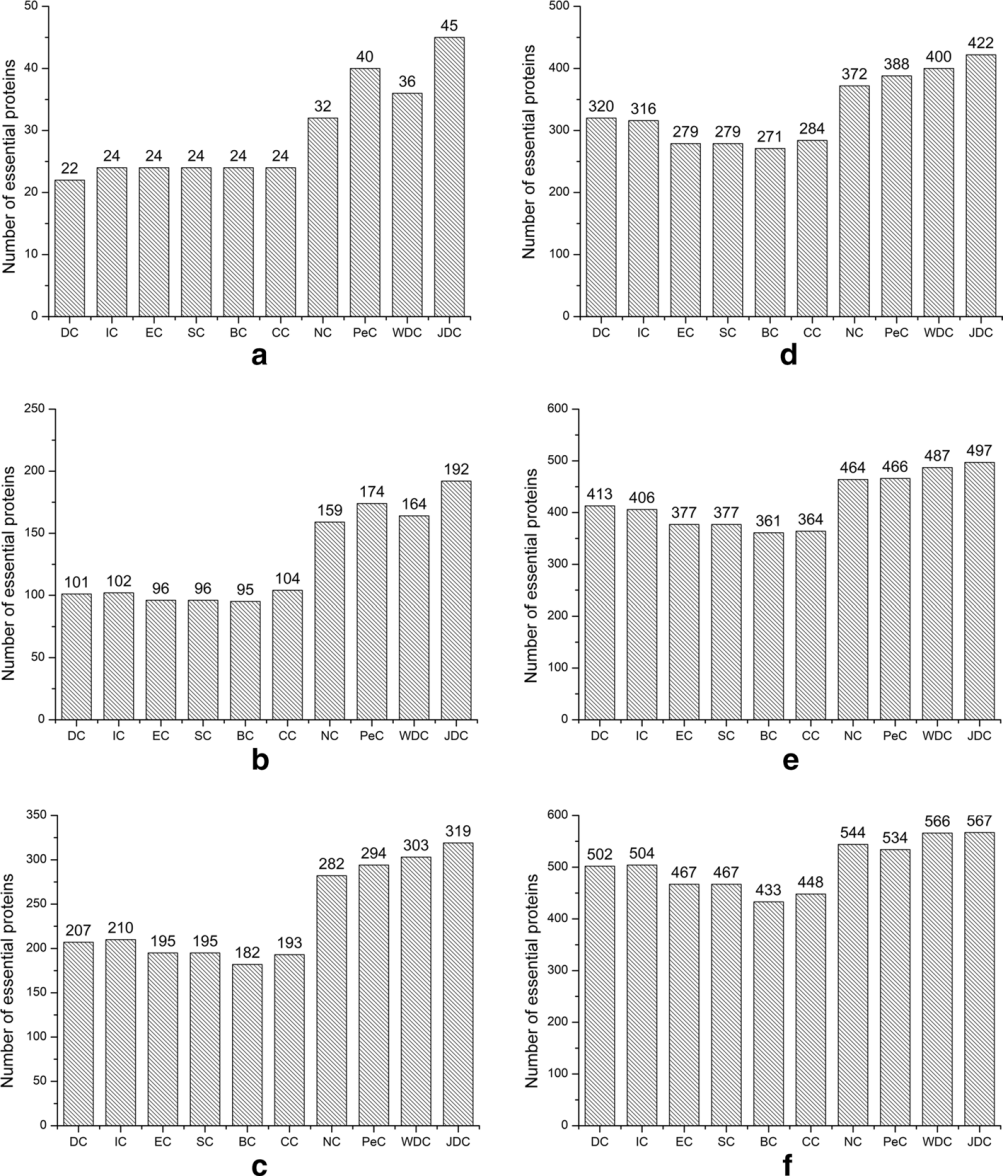
Compares the top 1%, 5%, 10%, 15%, 20% and 25% of essential proteins obtained by JDC with other methods in yeast data. **a** TOP1%. **b** Top5%. **c** Top10%. **d** Top15%. **e** Top20%. **f** Top25%

**Fig. 5:**
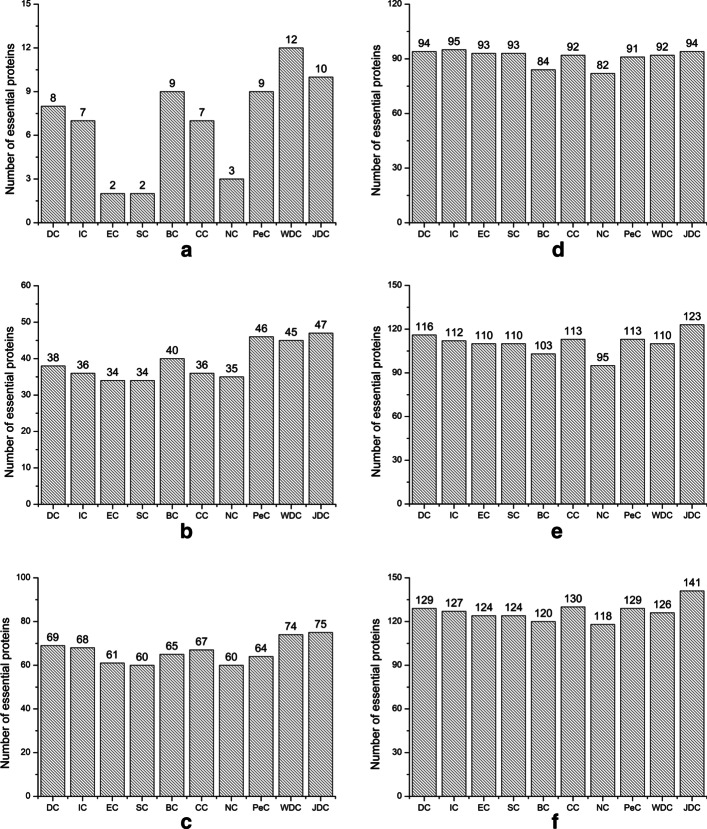
Compares the top 1%, 5%, 10%, 15%, 20% and 25% of essential proteins obtained by JDC with other methods in E.coli data. **a** TOP1%. **b** Top5%. **c** Top10%. **d **Top15%. **e** Top20%. **f** Top25%

To further validate the performance of JDC, we adopt a top analysis metrics that select the scores of each top percentage (top1%, top5%, top10%, top15%, top20%, top25%) of the methods and determine how many of these are essential proteins. The experimental results are shown in Figs. 4 and 5.

As shown in Fig. 4a of Yeast data, when we select the top 1% ranked proteins, JDC and other methods (DC, IC, EC, SC, BC, CC, NC, PeC, and WDC) identify 45, 22, 24, 24, 24, 24, 32,40 and 36 essential proteins, respectively. In the Yeast data, the JDC method can identify 45 essential proteins when we select the top 1% ranked proteins. Compared with the centrality method, the number of essential proteins that JDC can identify has increased by at least 43%. When compared with PeC and WDC, JDC can also improve by 12.5% and 25%, respectively. In Fig. 5, JDC can identify 10, 47, 75, 94, 123 and 141 essential proteins in each top percent (1%, 5%, 10%, 15%, 20% and 25%) of proteins on E.coli data. This shows that the JDC method is better than other methods at 5%, 10%, 20% and 25%.

To find the difference and overlap of essential proteins identified by each method, we select the top 100 proteins sorted by each method in yeast data, and investigate the overlapping relationships. Table 2 shows the intersection, difference of results between JDC and other various methods, and lists corresponding number and proportion of non-essential and essential proteins.

**Table 1:** SN, SP, FPR, PPV, NPV, F-measure, ACC and MCC of Various Methods on Total Ranked Proteins

Methods	SN	SP	FPR	PPV	NPV	F- measure	ACC	MCC
*Yeast data*								
JDC	**0.4604**	**0.8403**	**0.1597**	**0.4604**	**0.8403**	**0.4604**	**0.7535**	**0.3007**
DC	0.4002	0.8217	0.1783	0.4002	0.8217	0.4002	0.7251	0.2219
BC	0.3505	0.8069	0.1931	0.3505	0.8069	0.3505	0.7023	0.1574
CC	0.3548	0.8082	0.1918	0.3548	0.8082	0.3548	0.7043	0.163
SC	0.3676	0.812	0.188	0.3676	0.812	0.3676	0.7102	0.1796
EC	0.3676	0.812	0.188	0.3676	0.812	0.3676	0.7102	0.1796
IC	0.401	0.822	0.178	0.401	0.822	0.401	0.7255	0.223
NC	0.4353	0.8321	0.1679	0.4353	0.8321	0.4353	0.7412	0.2674
PeC	0.4036	0.8227	0.1773	0.4036	0.8227	0.4036	0.7267	0.2263
WDC	0.4576	0.839	0.161	0.458	0.8388	0.4578	0.7516	0.2967

Methods	SN	SP	FPR	PPV	NPV	F-measure	ACC	MCC
*E.coli data*								
JDC	**0.2835**	**0.9264**	**0.0736**	**0.2835**	**0.9264**	**02,835**	**0.8665**	**02,099**
DC	0.2559	0.9236	0.0764	0.2559	0.9236	0.2599	0.8614	0.1795
BC	0.2441	0.9224	0.0776	0.2441	0.9224	0.2441	0.8592	0.2665
CC	0.2441	0.9224	0.0776	0.2441	0.9224	0.2441	0.8592	0.1665
SC	0.2283	0.9207	0.0793	0.2283	0.9207	0.2283	0.8562	0.1491
EC	0.2283	0.9207	0.0793	0.2283	0.9207	0.2283	0.8562	0.1491
IC	0.2559	0.9236	0.0764	0.2559	0.9236	0.2559	0.8614	0.1795
NC	0.2165	0.9195	0.0805	0.2165	0.9195	0.2165	0.8541	0.1361
PeC	0.2441	0.9204	0.0776	0.2441	0.9224	0.2441	0.8592	0.1665
WDC	0.2689	0.922	0.078	0.2689	0.922	0.2689	0.859	0.1909

Where JDC **∩**${{\varvec{C}}}_{{\varvec{i}}}$ denotes the number of overlapping proteins identified by various prediction methods, and |${{\varvec{C}}}_{{\varvec{i}}}$**-**JDC| denotes the number of non-overlapping proteins identified by JDC and various centrality measures. As can be seen from Table 2, the number of non-essential proteins in JDC is smaller than that of other methods, and the proportion of essential proteins is much higher than that of other methods. Take BC as an example. The number of BC in |${{\varvec{C}}}_{{\varvec{i}}}$-JDC| is 85. The percentage of essential proteins of BC in |${{\varvec{C}}}_{{\varvec{i}}}$-JDC| was 42.35%, while JDC identified 78.82% essential proteins. This means that JDC can identify more essential proteins that BC is not.

### Jackknife analysis

Holman et al. devised a jackknife strategy that tests the performance of ranking methods [47]. We also use this method to evaluate the JDC method and other nine essential protein prediction methods. For each prediction method, we assess the performance by calculating the sum of the true essential proteins and the number of essential proteins. Figure 3 is the jackknife curve of various methods.

**Fig. 6:**
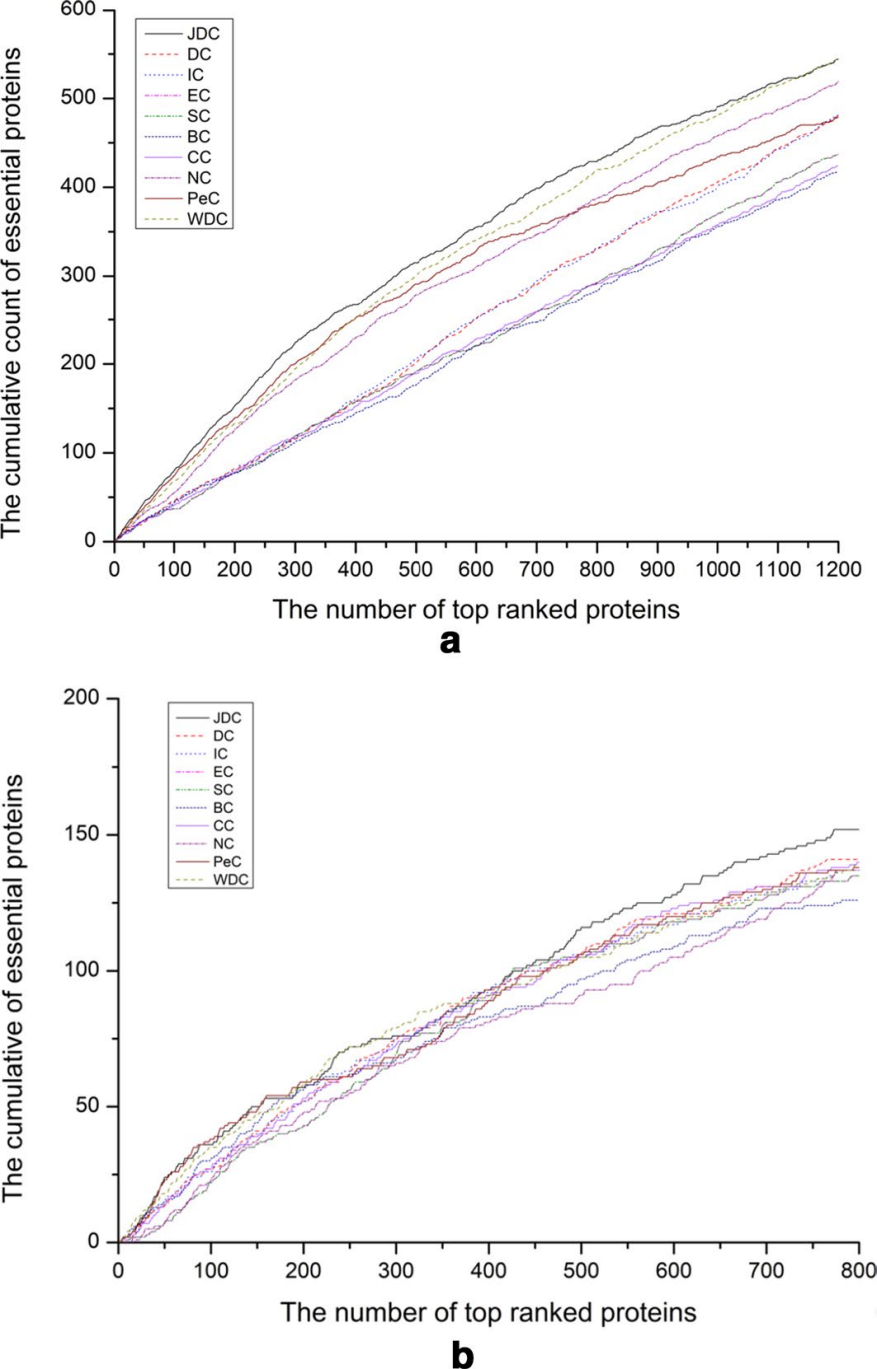
Jackknife curve of various prediction methods. **a** Yeast data. **b** E.coli data

The jackknife curve of ten essential protein prediction methods is plotted in Fig. 6. Where the vertical axis represents the cumulative count of essential proteins, and the horizontal axis represents the predicted number of essential proteins. The jackknife curve of the JDC method is higher than that of other nine methods (DC, IC, EC, SC, BC, CC, NC, WDC, and PeC). The results from the jackknife analysis show that the performance of JDC is superior to other prediction methods in identifying essential proteins. The advantage of JDC is that it can overcome the volatility of the gene expression data.

### Modularity analysis

**Fig. 7:**
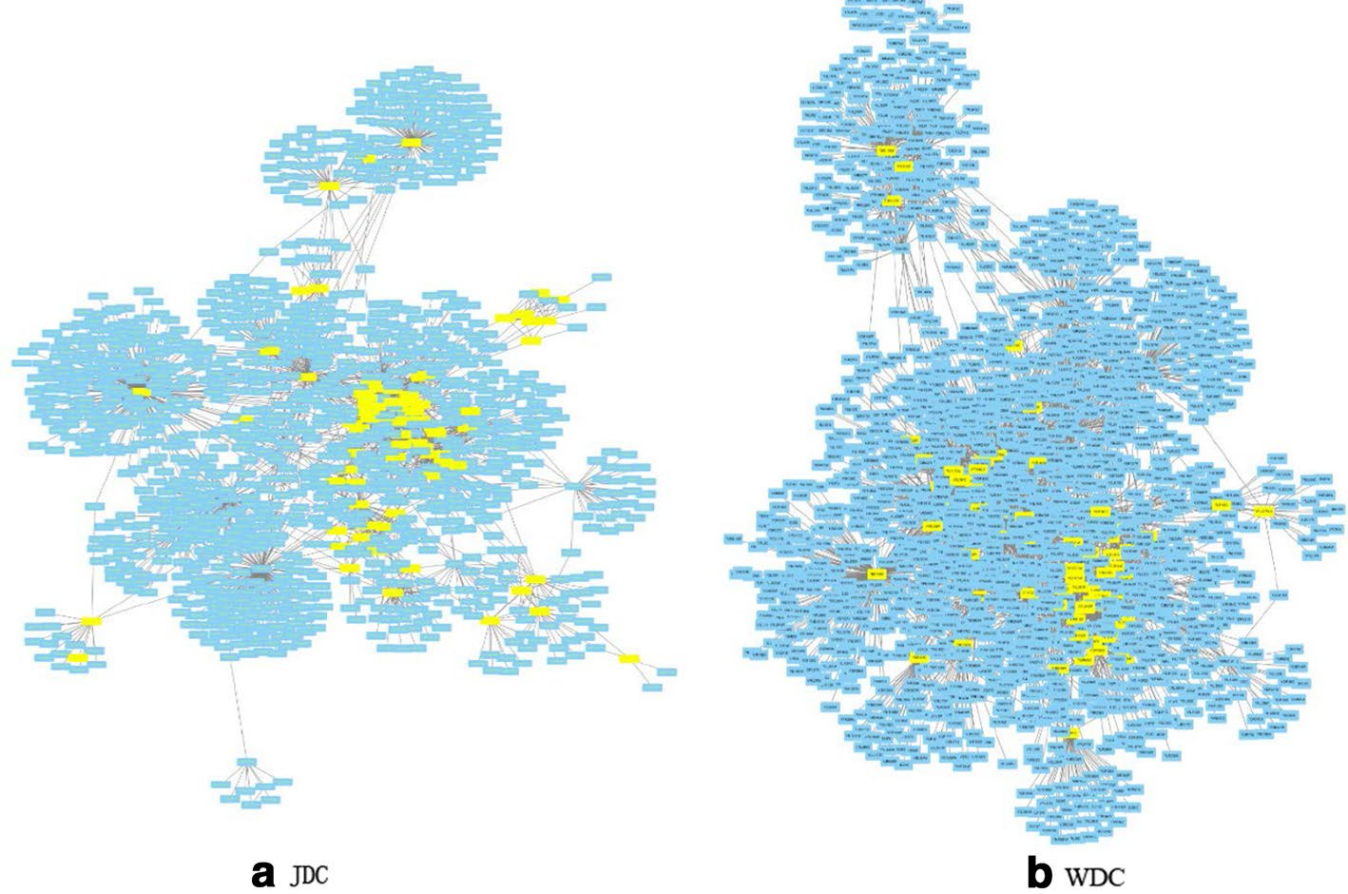
The modularity of interactions among the top 100 essential proteins predicted by JDC and WDC

Hart et al. indicate that the importance of proteins is not related to themselves, but specific protein complexes [48]. Zotenko et al. further demonstrate that functional protein modules contain a large number of essential proteins [49]. To verify the conclusion, we select the top 100 proteins ranked by JDC, and constructed a small PPI network module with those proteins and their neighbor proteins. The result is shown in Fig. 7. The top 100 proteins of JDC include 80 essential proteins (yellow nodes in Fig. 7a) and 17 functional modules by Markov Cluster procedure (MCL) [50]. For WDC, we follow a similar analysis as above, 68 essential proteins (yellow nodes in Fig. 7b)and 14 functional modules are found. The modularity of JDC presents more obvious than that of WDC. Besides, most of the essential proteins are hubs in the network, as shown in Fig. 7a, which is consistent with views of He et al. [51]. To compare the functional modules, we adopt the GO enrichment analysis by using website(http://geneontology.org/). By using JDC method, 11 out of 17 functional modules have p-value less than 0.05, whereas, 6 out of 14 functional modules with WDC have p-value less than 0.05.

### Results using fly and human dataset

To further prove the advantage of our method, we compare JDC with PeC and WDC methods on other two organisms: Fly and Human. The gene profiles for human are RNA-seq expression with tissue-specific labels, we select the two kinds of tissues dataset for further analysis. The results using Fly and Human datasets are listed in Table 3, which show the number of essential proteins in top 100, 200, 300, 400, 500, 600 essential candidates ranked by JDC, Pec and WDC. It can be found that the JDC almost presented the high-performance in the results, which indicate that the JDC had improvement over the other methods based on different organisms.

**Table 2:** The overlapping relationships between JDC and nine other prediction measures for the top 100 proteins

Centrality	$\mathrm{JDC}$∩${\mathrm{C}}_{\mathrm{i}}$	Non-essential proteins of ${\mathrm{C}}_{\mathrm{i}}$ in $\left|{\mathrm{C}}_{\mathrm{i}}-\mathrm{JDC}\right|$	Non-essential proteins of $\mathrm{JDC in }\left|{\mathrm{C}}_{\mathrm{i}}-\mathrm{JDC}\right|$	Percentage of essential proteins of ${\mathrm{C}}_{\mathrm{i}}$ in $\left|{\mathrm{C}}_{\mathrm{i}}-\mathrm{JDC}\right|$ (%)	Percentage of essential proteins of JDC in $\left|{\mathrm{C}}_{\mathrm{i}}-\mathrm{JDC}\right|$ (%)
DC	16	46	15	45.24	82.14
IC	17	46	18	44.58	78.31
EC	8	61	18	33.70	80.43
SC	8	61	18	33.70	80.43
BC	15	49	18	42.35	78.82
CC	13	52	17	40.23	80.46
NC	36	34	14	46.88	78.13
PeC	67	12	8	63.64	75.76
WDC	55	20	12	55.56	73.33

### Comparison with dynamic network framework

In the previous description, we compared JDC with various essential protein prediction methods that are proposed base on the static PPI network. The experimental results show that our method can improve the accuracy of essential protein prediction. To further prove the advantage of our method, we compare it with some methods that are designed based on the dynamic PPI network. We compare JDC with both NF-PIN and TS-PIN methods. The two existing methods, which use gene expression on yeast data, predict essential proteins in dynamic PPI networks. The results are shown in Tables 4 and 5.

**Table 3:** Accurate analysis of the number of essential proteins predicted by JDC, PeC and WDC on Fly and Human network

	Methods name	Top100	Top200	Top300	Top400	Top500	T600
Fly	JDC	**48**	**65**	**69**	**75**	79	85
	PeC	46	52	58	66	70	73
	WDC	43	64	68	73	**82**	**88**
Human Colon	JDC	93	**185**	**278**	**360**	438	**523**
	PeC	**94**	182	272	357	**445**	522
	WDC	87	178	271	355	435	512
Human Liver	JDC	**93**	**183**	**267**	**354**	437	**517**
	PeC	**93**	176	**267**	352	**438**	516
	WDC	83	171	258	345	430	509

**Table 4:** Accurate analysis of the number of essential proteins predicted by various central methods in the dynamic network of NF-PIN with JDC

Centrality	Top100	Top200	Top300	Top400	Top500	T600	Exceed times
JDC	80	**153**	**224**	**267**	**315**	**355**	**5**
NF-DC	55	111	167	221	261	303	0
NF-EC	55	110	157	202	239	276	0
NF-SC	55	116	161	204	239	276	0
NF-BC	50	97	133	188	226	254	0
NF-CC	45	87	122	161	193	230	0
NF-IC	55	111	167	221	261	303	0
NF-LAC	**82**	141	198	243	280	322	1
NF-NC	80	147	197	252	290	324	0

**Table 5:** Accurate analysis of the number of essential proteins predicted by various central methods in the dynamic network of TS-PIN with JDC

Centrality	Top100	Top200	Top300	Top400	Top500	T600	Exceed times
JDC	80	**153**	**224**	**267**	**315**	**355**	**5**
TS-DC	71	143	198	250	297	347	0
TS-EC	71	143	209	259	300	334	0
TS-SC	78	144	210	266	308	351	0
TS-BC	55	117	165	215	252	287	0
TS-CC	55	114	173	221	273	326	0
TS-IC	71	143	198	247	297	347	0
TS-LAC	**85**	138	196	246	300	350	1
TS-NC	82	142	200	253	301	350	0

The methods with dynamic PPI network can effectively improve the accuracy of the identification of essential proteins in DC, EC, SC, BC, CC, IC, LAC, and NC. As shown in Table 4, when the top100, top200, top300, top400, top500, and top600 proteins are selected, JDC can identify 80, 153, 224, 267, 315, and 355 essential proteins, respectively. As can be seen from Table 4, our method is better than that of other prediction methods at the top 200, top 300, top 400, top 500, and top 600. compared with the TS-PIN, which incorporated subcellular localization information, our method also has similar results. As shown in both Tables 4 and 5, the exceed times of our method are 5 and 5 respectively, which indicate the JDC method is an effective prediction method for essential proteins.

## Discussion

The difference between JDC and PeC or WDC is how to weight the PPI network. PeC and WDC both adopt the Pearson product-moment correlation coefficient to measure the similarity between two sets of gene expression values. However, the gene expression data can be represented with continuous values, which are prone to fluctuations that may affect prediction performance. JDC incorporate the Boolean values to represents the "on/off" state of genes at different times in biological development, and adopt Jaccard similarity index to measure the similarity between genes. JDC can fully consider the co-expression state of the connected genes at multiple different moments, while WDC and Pec compare the similarity of the specific expression values of the two genes at different times. Based on the results form Figs. 2 and 3, the ROC curve for JDC can almost achieve the best on the yeast dataset, and when values of FPR are less than 0.4 on the E.coli dataset, the ROC curve of JDC also has the similar results. The results suggest that the JDC has better sensitivity than that of WDC and PeC.

Recently, some computational methods for essential proteins prediction have been proposed, which employ a variety of biological data including sequence, orthology, evolution, expression, and subcellular localization information. We have further compared the JDC with recent developed methods for predicting essential proteins by using multiple biological information.

SPP adopts a strategy of sub-network partition and prioritization to predict essential proteins by fusing PPI network and subcellular localization data, which can identify 84, 153, 210, 261, 314, 362 essential proteins with different top set, respectively. Compare with SPP, the results of JDC are improved by 6.25%, 2.32%, and 0.32% in top 300, 400, 500 essential candidates, respectively. In top 100 and 600, SPP generates better results than that of JDC. The results indicate that both subcellular localization data and gene expression data can often improve the accuracy of essential protein prediction. NCCO fuses the PPI network and orthology information to predict the essential proteins, which integrate NCC (Neighborhood Closeness Centrality) and OS (Orthologous Scores). Compare with NCC, the result of JDC is better than that of NCC. Orthology information is adopted to assessed the conservative property of proteins. Many essential proteins of Yeast are conserved comparing with non-essential proteins, so OS is useful feature for NCCO to predict the essential proteins. NCCO exhibits the higher accuracy than the JDC. RWEP uses the random work algorithm to identify essential proteins by fusing PPI network and biological properties including subcellular localization information, gene expression, complex information, and GO annotation information. Comparing with RWEP, JDC achieved the better result at top 1%, the optimal results of RWEP are better than that of JDC at top 5%-20%. In order to get the optimal results, RWEP adopts a parameter to adjust the contribution of proteins’ own scores and their neighbors’ scores, which is a need to tune the parameters, however it is difficult to choose the best parameters for different datasets. Different parameters have a great influence on the experimental results. In summary, fusing more biological data can improve the effectiveness of methods to identify essential proteins.

## Conclusions

In this study, we propose a new essential protein recognition algorithm named JDC based on the PPI networks and gene expression data. JDC eliminates the influences of fluctuations in gene expression data by calculating the similarity of "active" and "inactive" state of gene expression in a cluster of the PPI network. Compared with the nine prediction methods using static PPI network and two dynamic prediction methods, JDC is an effective essential protein prediction method. As future work, it would be more accurate to predict essential proteins by further utilizing the time-series gene expression dataset. For the time series data, the dynamic methods can be used to refine the PPI network to construct a reliable PPI network, and a method can be revised to segment the time series data, and within each segment to construct a static network with binarizing gene expression data. The new method would be considered both advantages of dynamic network methods and the JDC method.

## Data Availability

The data and source codes are available in https://github.com/jczhongcs/JDC

## Abbreviations

PPI: Protein–Protein interaction
DC: Degree Centrality
BC: Betweenness Centrality
CC: Closeness Centrality
SC: Subgraph Centrality
EC: Eigenvector Centrality
IC: Information Centrality
ECC: Edge clustering coefficient
ROC: Receiver Operating Characteristic
AUC: Area Under receiver operating characteristic Curve
SN: Sensitivity
SP: Specificity
FPR: False-Positive Rate
PPV: Positive Predictive Value
NPV: Negative Predictive Value
ACC: Accuracy
MCC: Matthews correlation coefficient
MCL: Markov Cluster procedure; ORF, ST, PHY
